# Investigation of Flow Characteristics in a Stirred-Tank Bioreactor with Flexible Blades via Integrated PIV and Image Recognition

**DOI:** 10.3390/bioengineering13040415

**Published:** 2026-04-01

**Authors:** Wenda Xu, Chengfan Cai, Zhe Li, Hancheng Lu, Chao Yang, Baoqing Liu

**Affiliations:** 1 Institute of Advanced Equipment, Zhejiang University, Hangzhou 310027, China; wendaxu@zju.edu.cn (W.X.); lizheee@zju.edu.cn (Z.L.); hanchenglu@zju.edu.cn (H.L.); a1402059002@126.com (C.Y.); 2 Institute of Wenzhou, Zhejiang University, Wenzhou 325006, China; wzcaichengfan@163.com

**Keywords:** flow characteristics, flexible blade, Particle Image Velocimetry, image recognition, stirred-tank bioreactor

## Abstract

Biological reactions are widely applied in processes such as bioenergy production, raw material manufacturing, and resource recovery from waste. As a main reactor type, the stirred-tank bioreactor exhibits prominent advantages of high mixing efficiency and strong adaptability. At present, the optimization of bioreactors mainly focuses on rigid impellers, and the research on flexible impellers is insufficient. Identifying the influence of flexible materials on bioreactor performance is of great significance. In this work, a stirred-tank bioreactor equipped with flexible blades was designed. In addition, a performance detection method coupling Particle Image Velocimetry (PIV) and image recognition was proposed to systematically study the effects of stirring speed, liquid environment, and impeller type. The results indicated that compared with rigid impellers, flexible impellers could reduce 7.7% low-velocity zones and save 15% mixing time. Velocity could be distributed more uniformly, and the suitable velocity ratio was increased by 7.88%. Moreover, the power consumption had been reduced by 7.49%. Taking into account the mixing efficiency and the impact of shear stress, the optimized structural combination and operating parameters were a pitched blade turbine (PBT)-propeller impeller type and a stirring speed of 300 rpm. This work provides important references for the design and optimization of stirred-tank bioreactors.

## 1. Introduction

Biological reaction, as a transformation method centered on enzymes or cells, has broad application prospects. Compared with chemical reaction, it can proceed efficiently under mild reaction conditions, which typically means lower power consumption and equipment costs. In addition, biological reactions are usually environmentally friendly and sustainable [1,2]. With the intensification of global warming and extreme weather events, a growing number of countries have begun to commit themselves to reducing carbon emissions. Some technologies centered on biological reactions, such as bioenergy production [3,4], raw material manufacturing [5,6], and resource recovery from waste [7,8], have attracted widespread attention.

As the key equipment for biological reactions, the operational performance of bioreactors directly determines the mass transfer, heat transfer, and reaction efficiency. At present, the mainstream types of bioreactors include airlift bioreactors [9], membrane bioreactors [10], trickle bed bioreactors [11], stirred bioreactors [12], and so on. Among them, the stirred-tank bioreactor has the advantages of high mixing efficiency and strong adaptability. Its working efficiency is affected by the structural parameters of agitators, reactor vessels, and baffles. Xu et al. [13] developed a type of V-shaped perforated baffle (VSPB). Through macro-mixing experiments and computational fluid dynamics (CFD) simulations, it was found that the VSPB can significantly increase the tangential velocity and turbulent kinetic energy (TKE) of the fluid in the baffle region and effectively shorten the mixing time at low stirring speed. Yamada et al. [14] investigated the mixing performance of a rotating impeller (SWINGSTIR^®^) through decolorization experiments, ultrasonic velocity profiler (UVP) measurements, and CFD simulation. They found that increasing the blade width leads to a decrease in mixing performance, while increasing the rotating diameter can enhance the turbulence intensity and improve the mixing efficiency. Li et al. [15] designed an elliptical baffle-free stirred tank. Through experiments and numerical simulations, it was found that the new-type stirred tank suppresses the formation of central-surface vortices and columnar vortices via the sidewall extrusion effect. As a result, the mixing time is reduced by 10 to 15 times, and the TKE in the agitator region is increased by 2 to 3 times. Govara et al. [16] developed a new laboratory-scale bioreactor using a vibrating stirrer and a modified flask through the Box–Behnken design. This bioreactor can achieve intense mixing under low shear stress. However, most optimization studies are conducted using rigid impellers, while the research employing flexible impellers remains notably scarce. Furthermore, the influence of flexible blades on the flow characteristics in stirred-tank bioreactors still requires further elucidation.

Improving mixing efficiency usually relies on increasing stirring speed. However, increasing the speed will inevitably lead to a increase in the shear stress. For shear-sensitive microorganisms, high shear stress will affect their activity [17,18]. In addition, high power consumption is also a crucial factor restricting the development of stirred-tank bioreactors [19]. Against this background, a flexible impeller has been proposed. Its core advantage lies in the ability to deform under the action of fluid, thereby reducing mechanical damage to microorganisms. In addition, the flexible impellers can reduce the resistance directly acting on their surface, which can effectively lower the power consumption. Chen et al. [20] investigated the effects of flexible impellers on the flow characteristics and droplet distribution of O/W emulsions using experiment and CFD simulation. They found that flexible impellers can generate more uniform TKE, turbulent dissipation rate, and dispersed phase distribution, with a more concentrated droplet size distribution and a smaller Sauter mean droplet diameter. Gu et al. [21] constructed a CFD-PBM model to investigate the flow characteristics of stirred tanks equipped with rigid impellers, rigid–flexible combined impellers, and rigid–flexible combined perforated impellers. The results showed that a longer length of the flexible connecting component is more conducive to the gas–liquid dispersion. Liang et al. [22] experimentally studied the turbulent flow field in a stirred tank under the action of three impeller types (flexible impellers, flat rigid impellers, and curved rigid impellers). It was found that the vibration of the impeller blades disturbs the surrounding flow field and enhances the transport of TKE from the impeller blades to the main region of the vessel. Previous studies have demonstrated that flexible impellers possess certain advantages. But they have overlooked the impact of the presence of flexible blades on shear stress, while shear stress poses a threat to the survival of sensitive cells [23,24,25]. Our work considers a variety of parameters including shear stress and proposes a velocity zoning method to evaluate the effect of flexible impellers.

CFD simulation is a widely accepted research method that can reduce the cost of repeated experiments. However, existing simulations involving flexible impellers often approximate the blades as rigid bodies, which inevitably compromises some accuracy. By monitoring the actual flow characteristics inside the stirred tank, experimental measurements can better describe the fluid–structure interaction behavior. Therefore, we adopted the experimental approach in this work. As a non-intrusive optical measurement method, PIV has high resolution and accuracy, while avoiding the interference of traditional intrusive measurement methods on the flow field [26,27]. Laakkonen et al. [28] used PIV to study the flow characteristics of stirred tanks under two systems (air–water and carbon dioxide–n-butanol). They found that PIV can simultaneously measure parameters such as flow field, gas holdup, and gas–liquid interfacial area, providing support for the verification of stirring simulation results. Hoseini et al. [29] used PIV to measure the flow field, average velocity components, and kinetic energy of the system equipped with double finger-shaped baffles and a swept-blade agitator, verifying the accuracy of the IBM-LES model in studying unsteady turbulence in agitators with complex geometries. Neogi et al. [30] used PIV to study the flow field of three types of Geldart A particles (flaky, spherical, and irregular) in the free zone of a pilot-scale fluidized bed. They found that particle shape greatly affects the bed expansion ratio; eddies in the free board zone always exist but dissipate rapidly, and most streamlines are in a laminar flow state. He et al. [31] conducted a visualization study on the flow field in two types of cyclone separators: conventional and reverse cyclone (RC). They found that the tangential and radial velocity distributions of the RC increase at higher flow rates and decrease at lower heights, while the velocity distributions still maintain good symmetry and regularity. In summary, previous studies have fully validated the feasibility and robustness of PIV for flow field measurements. In view of this, our work employs PIV to capture and visualize the flow field, aiming to provide reliable experimental data for revealing the influence mechanism of flexible blades on the flow characteristics.

Through a comprehensive literature review, it is evident that there is an obvious gap in the optimization of stirred-tank bioreactors using flexible impellers, and existing studies have insufficiently addressed the influence of shear stress. To address this issue, a performance detection method based on PIV and image recognition is proposed to analyze the effects of flexible or rigid impellers on flow characteristics under different rotational speeds, liquid environment, and impeller types. This work validates the application potential of flexible impellers in bioengineering and provides valuable references for the design and optimization of stirred-tank bioreactors.

## 2. Experiments

### 2.1. Experimental Setup

**Figure 1:**
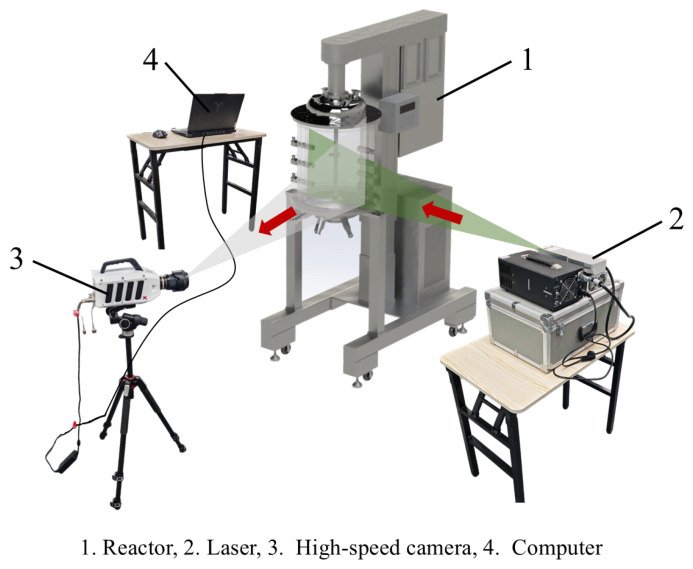
Sketch of the experimental system.

As shown in Figure 1, all experiments were conducted in a 30 L laboratory-scale stirred-tank bioreactor. The stirring speed was adjusted by a PLC control system, with an adjustable range of 50~1000 rpm. The PIV system consisted of a laser light source, a high-speed camera, and a computer. Parameters of experimental instruments are provided in Table 1.

During the experiment, the required concentration of the liquid environment was prepared. Glass microsphere tracer particles with a particle size of 10 μm and a density of 1.1 g/cm^3^ were added from the opening above the bioreactor. After stirring thoroughly for 5 min to ensure the tracer particles were evenly mixed in the liquid phase, subsequent experimental operations were carried out.

In this work, two impeller arrangements (single-layer and double-layer) were investigated. The single-layer arrangement employs only one impeller set fixed to the stirring shaft; by contrast, the double-layer arrangement comprises two discrete impeller assemblies spaced at a specified axial interval along the same shaft. For the single-layer arrangement, four configurations were adopted: rigid PBT (PBT-R), flexible PBT (PBT-F), rigid propeller (Propeller-R), and flexible propeller (Propeller-F). For the double-layer arrangement, two combined configurations were employed: rigid PBT–propeller (PBT–Propeller-R) and flexible PBT–propeller (PBT–Propeller-F).

**Figure 2:**
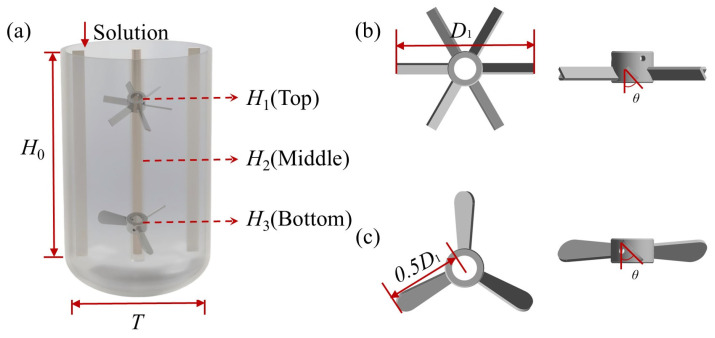
Structure of stirred-tank bioreactor: (**a**) Bioreactor. (**b**) Pitched blade turbine. (**c**) Propeller.

As shown in Figure 2a, taking the bioreactor equipped with double-layer impellers as the example, the experimental setup was a cylindrical transparent stirred tank with two baffles and an elliptical bottom. Its inner diameter *T* was 300 mm, its straight-side height *H*_0_ was 450 mm, and the depth from the bottom end of the stirring shaft to the tank bottom was *T*/3. Two baffles, which ran through the longitudinal section of the tank and had a width of *T*/12, were arranged at a position 0.05*T* away from the inner wall of the stirred tank to minimize the impact of surface eddies on the experiment. Considering that bioreactors in industry usually have a relatively high aspect ratio, double-layer impellers were selected and equipped. In this experiment, the heights *H*_1_, *H*_2_, and *H*_3_ of the three designed stirring impeller installation positions to the tank bottom were 0.53*T*, 0.83*T*, and 1.13*T*, respectively.

As shown in Figure 2b,c, the rigid impellers equipped in the bioreactor were a PBT and a propeller, which had an inclination angle *θ* of 45° and a diameter *D*_1_ of 0.45*T*. The width and thickness of the rigid PBT were 12 mm and 3 mm. The flexible impeller consisted of a flexible blade and a rigid hub, which were firmly fastened via bolts. The flexible blade performed the core stirring and mixing function, while the rigid hub was designed for stable assembly with the stirring shaft. In this work, the flexible blade was fabricated from stainless steel (with the density of 7.93 g/cm^3^, elastic modulus of 193 GPa and Poisson’s ratio of 0.3) with a length *l* = 0.1*T* and a thickness *d* = 1.5 mm. The rigid hub had a diameter *D*_2_ = 0.25*T*, and different impeller diameter ratios could be achieved by replacing flexible blades of varying lengths. The fundamental difference between flexible impellers and rigid impellers lies in the fluid–structure interaction effect. Unlike rigid impellers, which maintain a constant geometry and generate a steady flow field, flexible impellers undergo adaptive structural deformation in response to fluid loads, which can effectively reduce the shear stress and power consumption in the stirred tank.

In the experiment, to reduce the impact of reflections from metal surfaces during the measurement of flow characteristics, all metal surfaces that could be exposed to light were covered with black matte waterproof tape. In addition, for a single-layer impeller, the impeller was either PBT or propeller, while the installation position was *H*_2_.

Sodium carboxymethyl cellulose (CMC) is a commonly used food thickener. CMC solution is typical non-Newtonian fluid, exhibiting obvious shear-thinning characteristics [32]. The rheological properties of the CMC solution can be characterized by the power-law equation, as shown in Equations (1) and (2):

(1) $$\tau =K\cdot {\dot{\gamma }}^{n}$$

(2) $$\mu_{a}=\frac{\tau }{\dot{\gamma }}=K\cdot {\dot{\gamma }}^{n-1}$$

The experiment was conducted at 25 °C and standard atmospheric pressure, with water having the density of 997 kg/m^3^ and viscosity of 0.89 mPa·s. The consistency coefficient *K* of 0.5 wt% and 1.0 wt% CMC solutions was 0.167 kg/m·s^n−2^ and 0.232 kg/m·s^n−2^, and the flow behavior index *n* was 0.946 and 0.793, respectively.

In the experiment, CMC solutions with mass fractions *ω*_1_ of 0 wt% (water), 0.5 wt%, and 1 wt% were prepared as the liquid-phase environment. Six sets of stirring speed conditions were designed, including 150 rpm, 200 rpm, 250 rpm, 300 rpm, 350 rpm, and 400 rpm. The specific experimental operating conditions are shown in Table 2. The double-layer arrangement assembly consists of an upper PBT impeller and a lower propeller impeller, mounted at positions *H*_1_ and *H*_3_, respectively. The single-layer arrangement utilizes either a PBT impeller or a propeller impeller, installed at position *H*_2_ (the middle of *H*_1_ and *H*_3_).

### 2.2. Measurement Methods

#### 2.2.1. Measurement of Velocity Distribution

**Figure 3:**
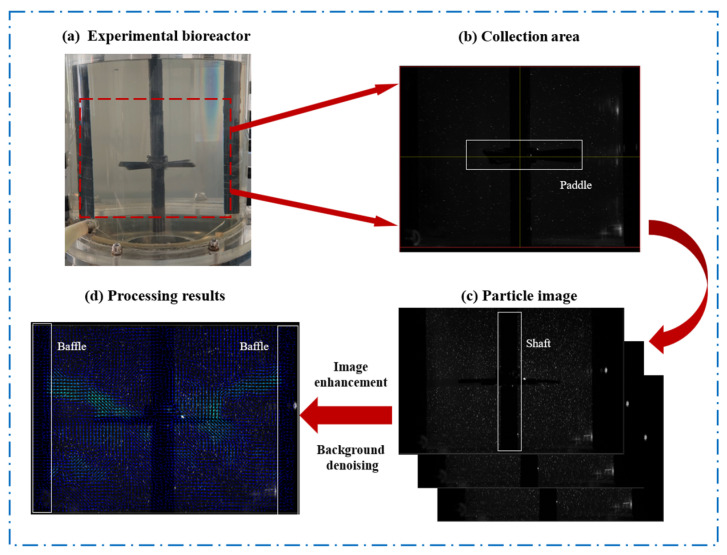
Principle of Particle Image Velocimetry.

As an advanced non-contact flow field measurement technology, PIV can simultaneously measure the velocity distribution on a cross-section and the three-dimensional velocity field in a flow field [33]. As shown in Figure 3, the basic principle of PIV is as follows: uniformly distributing tracer particles of a certain concentration in the flow field, illuminating the field with a laser light source, capturing images of particle movement using a high-speed camera, and finally processing the captured images of particle movement at different times.

In this experiment, a visual experimental system was set to measure the velocity distribution on the cross-section of the bioreactor. The acquisition frequency of the high-speed camera was 2000 Hz. The glass microbead tracer was added at a concentration of 0.15 g/L. The velocity of the tracer particles was calculated according to Equation (3) as follows:

(3) $$\left\{\begin{array}{l} v_{x}=\underset{dt\to 0}{\lim}\frac{dx\left(t\right)}{dt}\approx \frac{x\left(t+\Delta t\right)-x\left(t\right)}{\Delta t}\\ v_{y}=\underset{dt\to 0}{\lim}\frac{dy\left(t\right)}{dt}\approx \frac{y\left(t+\Delta t\right)-y\left(t\right)}{\Delta t}\\ v_{0}=\sqrt{v_{x}^{2}+v_{y}^{2}} \end{array}\right.$$

In this work, PIV was employed to separately measure the flow characteristics inside the bioreactor under the action of rigid impellers or flexible impellers. The pumping mode of the impellers was upward pumping. All measurements were performed when the flow field stabilized after 5 min of stirring.

#### 2.2.2. Measurement of Suitable Velocity Ratio

Image recognition identifies the color features of images through algorithms and correctly classifies the image content according to its respective category [34]. As the most fundamental and crucial information, the effective representation of color features relies on a scientific color space system. Currently, red, green, blue (RGB) and hue, saturation, value (HSV) are the two most widely used types of color spaces. RGB color space is based on the Cartesian coordinate system and represents colors using a linear combination of the red, green, and blue color components [35]. HSV color space uses hue, saturation, and value to represent colors. Compared with RGB, HSV can express the lightness, hue, and saturation of colors more intuitively, making it easier to compare different colors.

The improvement of mixing efficiency in bioreactors usually relies on increasing the stirring speed. However, the increase in stirring speed will inevitably lead to the enhancement of shear stress, which will have a significant impact on the sensitive cells. Against this background, the suitable velocity ratio has been proposed to comprehensively evaluate the mixing efficiency and the influence of shear stress. The measurement of the suitable velocity ratio is based on image recognition, which is obtained by extracting the color proportion corresponding to the appropriate speed range. Extract the RGB color space of the velocity contour, and convert the *R*, *G*, and *B* values to the range of 0~1 according to Equation (4) as follows:

(4) $${\left(R,G,B\right)}^{\prime }=\frac{\left(R,G,B\right)}{255}$$

Convert the obtained (*R*, *G*, *B*)′ values to *H*, *S*, and *V* values according to Equations (5)–(7) as follows:

(5) $$V=\max{\left(R,G,B\right)}^{\prime }$$

(6) $$S=\max{\left(R,G,B\right)}^{\prime }-\min{\left(R,G,B\right)}^{\prime }$$

(7) $$H=\left\{\begin{array}{ll} 0\left(if\;S=0\right)\\ \frac{60\left(G-R\right)}{S}+60 & \left(if\;V-S=B\right)\\ \frac{60\left(B-G\right)}{S}+180 & \left(if\;V-S=R\right)\\ \frac{60\left(R-B\right)}{S}+300 & \left(if\;V-S=G\right) \end{array}\right.$$

Based on the obtained *H*, *S*, and *V* values, set the minimum and maximum values for the target color. By creating a binarized image, the target region is displayed as white, while all other regions are displayed as black. Then, extract the proportion of the target color in the velocity contour, and calculate to obtain the suitable velocity ratio.

#### 2.2.3. Measurement of Mixing Time

Mixing time (*t*) was used to characterize the mixing effect of different impellers, and its measurement adopted the color-change method [36]. In the bioreactor filled with liquid phase, an iodine solution with a mass fraction *ω*_2_ of 30 wt% (prepared in a ratio of I_2_:KI = 1:2) was added, followed by thorough stirring until the solution was colored into a uniform and stable brown. Then, a slightly excessive amount of sodium thiosulfate solution with a mass fraction *ω*_3_ of 27 wt% was continuously added to the bioreactor to conduct a visualized decolorization experiment. The prepared solutions were injected into the bioreactor via the draft tube arranged near the left baffle. A high-definition camera was used to record the changes in the flow field structure inside the bioreactor, as well as the entire coloring and decolorization processes. The mixing time was measured in triplicate in parallel, and the average value was taken to reduce errors.

#### 2.2.4. Measurement of Power Consumption

Input power (*P*) is a common evaluation indicator in stirring operations. The torque value was measured using the torque method, and then *P* was calculated through formula derivation. *P* was calculated according to Equation (8) as follows:

(8) $$P=\frac{2\pi N\left(M_{1}-M_{0}\right)}{60}$$

In the experiment, measurements were taken 3 times and the average value was used to reduce errors. The power consumption per unit volume (*P*_V_) was the input power *P* divided by the working volume (*V*_0_). *P*_V_ was selected to evaluate the power consumption under different working conditions.

#### 2.2.5. Measurement of Shear Stress

The local shear stress is derived through the coupling of the velocity gradient field and rheological parameters (power-law equation) [37].

The radial and axial velocities (*u_r_* and *u_z_*) of the tracer particles are acquired via the PIV system, while the corresponding radial and axial coordinates (*r* and *z*) are acquired through structured discretization of the computational domain. The shear rate components can be written as follows:

(9) $$\left\{\begin{array}{l} {\dot{\varepsilon }}_{rr}=2\frac{\partial u_{r}}{\partial r}\\ {\dot{\varepsilon }}_{zz}=2\frac{\partial u_{z}}{\partial z}\\ {\dot{\varepsilon }}_{\theta \theta }=2\frac{u_{r}}{r}\\ {\dot{\gamma }}_{rz}=\frac{\partial u_{r}}{\partial z}+\frac{\partial u_{z}}{\partial r} \end{array}\right.$$

Based on the above four datasets, the shear rate can be calculated as follows:

(10) $$\dot{\gamma }=\sqrt{2{\left(\frac{\partial u_{r}}{\partial r}\right)}^{2}+2{\left(\frac{\partial u_{z}}{\partial z}\right)}^{2}+2{\left(\frac{u_{r}}{r}\right)}^{2}+{\left(\frac{\partial u_{r}}{\partial z}+\frac{\partial u_{z}}{\partial r}\right)}^{2}}$$

Therefore, the shear stress can be determined using Equation (1), based on the obtained shear rate and rheological parameters.

## 3. Results and Discussion

### 3.1. Velocity Distribution

In this section, the velocity distributions of rigid impellers and flexible impellers under different stirring speeds and different liquid concentrations are studied. Li et al. [38] indicated that chaotic mixing zones and mixing isolation zones exist during the agitation of shear-thinning non-Newtonian liquids. The existence of mixing isolation zones exerts a significant impact on mixing efficiency. Refer to the definition of mixing isolation zones: regions where the velocity is relatively low, creating a velocity difference with the surrounding areas, are defined as low-velocity zones. In this work, the low-velocity zone is the region where the velocity is less than 0.125 m/s.

**Figure 4:**
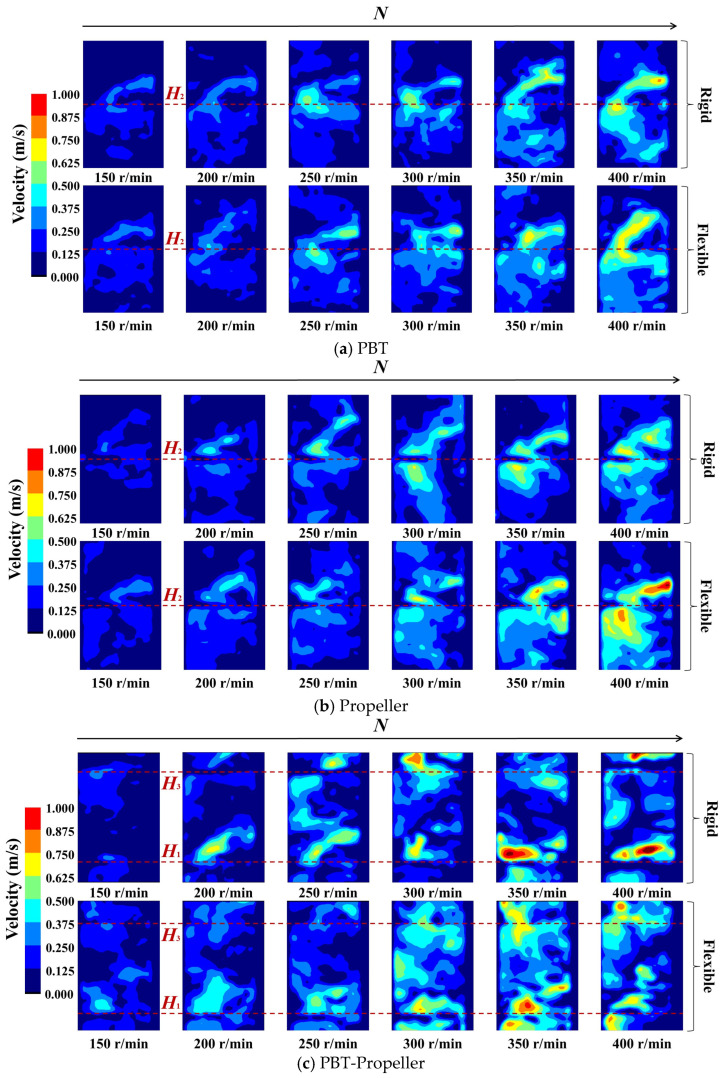
Velocity distribution in the bioreactor (*ω*_1_ = 0 wt%).

As shown in Figure 4, when *ω*_1_ is 0 wt%, the pumping effect of the stirring impeller is obvious. As the stirring speed increases, both the peak values of the velocity distribution of the three groups of impellers and their radial coverage show an increasing trend.

As shown in Figure 4a,b, for a single-layer impeller PBT or propeller, an obvious axial circulation is formed at the installation position of the stirring impellers, and as the stirring speed increases, the effect of axial circulation becomes more significant. When the stirring speed is low, there are low-velocity zones near the upper part of the stirring shaft, as well as at the liquid surface and the bottom of the bioreactor. With the increase in stirring speed, the low-velocity zones at the liquid surface and the reactor bottom are significantly eliminated, while the low-velocity zone near the upper part of the stirring shaft still exists. Under the action of PBT, the flow field has lower peak velocity, more uniform velocity distribution, and a wider radiation range. Under the action of propeller, the flow field has a significantly higher velocity peak and a more concentrated velocity distribution. This flow pattern is consistent with findings from previous studies. Gao et al. [39] investigated the flow characteristics of PBT via CFD simulations, observing the formation of distinct recirculating vortex structures in the vicinity of the blades. The high-velocity region is concentrated near the impeller, with the velocity magnitude gradually attenuating along the radial and axial directions away from the blades. Furthermore, the low-velocity regions are also observed at the liquid surface and the reactor bottom. The high degree of consistency in the flow structures obtained from both experiments and simulations validates the reliability and accuracy of the PIV system.

As shown in Figure 4c, for the double-layer impeller PBT-propeller, axial circulation is formed at the installation positions of both impellers. The low-velocity zone exists at the central position of the impellers, and when stirring speed increases to 250 rpm, the low-velocity zone is almost eliminated. Compared with a single-layer impeller, the configuration of double-layer impellers can effectively eliminate low-velocity zones, but the aggregation effect of velocity peaks becomes more pronounced.

For the same group of impellers, compared with rigid impellers, flexible impellers can better weaken the aggregation effect of velocity distribution and distribute kinetic energy more evenly throughout the bioreactor, and this improvement effect is more significant at lower stirring speeds (*N* ≤ 300 rpm). The low-velocity zones can be reduced by up to 23.8%, with an average reduction of 9.9%. This may be because flexible impellers can undergo elastic deformation according to the pressure they bear. Non-Newtonian fluids exhibit shear-thinning characteristics, meaning the higher the stirring speed, the lower the viscosity of the liquid phase. Flexible blades undergo adaptive deformation at different stirring speeds, thus being able to better disperse kinetic energy throughout the entire bioreactor.

**Figure 5:**
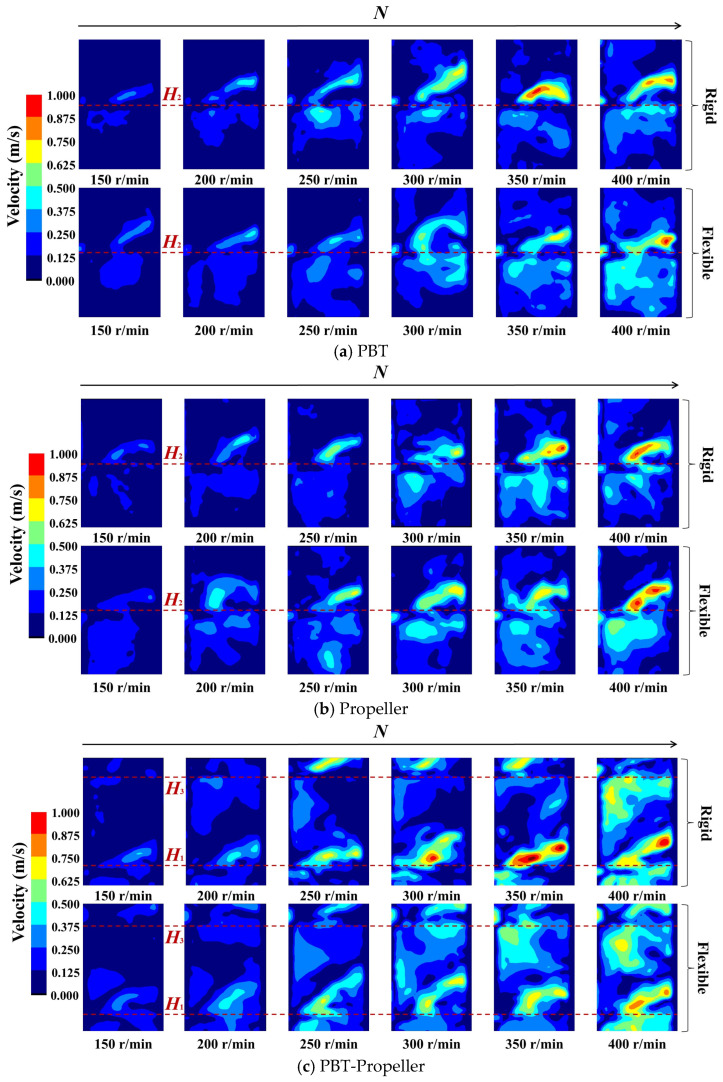
Velocity distribution in the bioreactor (*ω*_1_ = 0.5 wt%).

As shown in Figure 5, when *ω*_1_ is 0.5 wt%, the disturbance range of the stirring impeller at the same stirring speed decreases significantly. As the stirring speed increases, the velocity distribution shows the same trend as before (when *ω*_1_ is 0 wt%): the velocity peaks and radial coverage of the three groups of impellers all increase. But compared with the low concentration, the magnitude of the increase is significantly reduced.

As shown in Figure 5a,b, for a single-layer impeller PBT or propeller, the axial circulation at the installation positions of the stirring impellers is significantly weakened. The range of low-velocity zones above the stirring shaft and at the liquid surface of the bioreactor has expanded. Compared with that when *ω*_1_ is 0 wt%, as the stirring speed increases to 300 rpm, the PBT impeller can still diffuse the kinetic energy to the entire cross-section. However, the propeller shows a disadvantage in terms of dispersion effect, and when the stirring speed increases to 400 rpm, the low-velocity zone still exists.

As shown in Figure 5c, for the double-layer impeller PBT-propeller, the axial circulation effect of the upper PBT impeller is weakened more significantly. The low-velocity zone exists almost entirely in the middle and upper layers of the bioreactor. When the stirring speed increases, both the radial coverage and dispersion effect of the PBT impeller are significantly improved. In contrast, under the action of the propeller, the velocity peak increases significantly, while the dispersion range does not show an obvious expansion.

When *ω*_1_ increases from 0 wt% to 0.5 wt%, the effectiveness of the three groups of impellers is significantly weakened. But at the same stirring speed, flexible impellers still exhibit a better dispersion effect compared with rigid impellers. The maximum reduction in low-velocity zones can reach 26.5%, while the average value has dropped to 9.3%. For the PBT, flexible impellers are the first to form a complete axial circulation at the stirring speed of 300 rpm. For the propeller, flexible impellers can weaken the aggregation effect of kinetic energy. For the PBT-propeller, flexible impellers have an obvious advantage in eliminating low-velocity zones.

**Figure 6:**
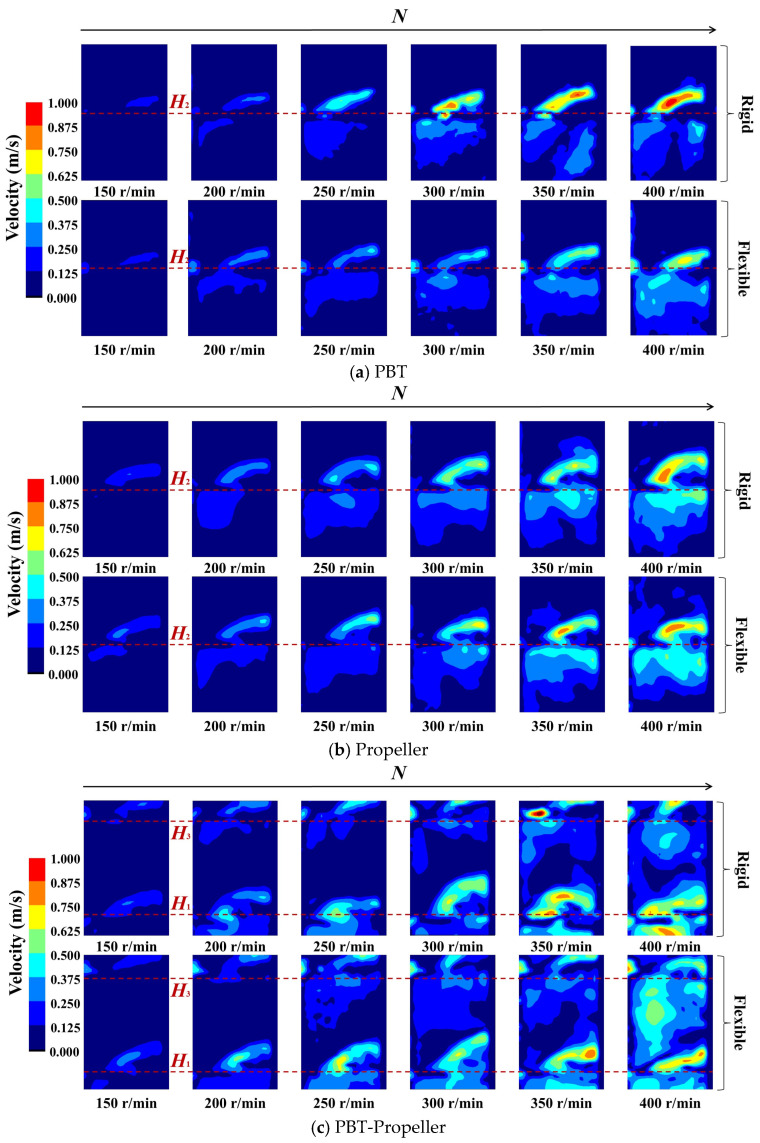
Velocity distribution in the bioreactor (*ω*_1_ = 1 wt%).

As shown in Figure 6, when *ω*_1_ is 1 wt%, the axial circulation effect of the stirring impeller is further weakened. When the single-layer impeller is in operation, the low-velocity zone always exists. However, when the stirring speed of the double-layer impeller reaches 300 rpm, 50% of the low-velocity zones are eliminated. This demonstrates the advantages of multi-layer impellers in bioreactors with a high aspect ratio.

At high concentrations, flexible impellers still have an advantage over rigid impellers in terms of kinetic energy dispersion. But this advantage is significantly weakened compared to the scenario when *ω*_1_ is 0 wt% or 0.5 wt%. The maximum reduction in low-velocity zones is 20.5%, while the average reduction is only 3.8%. This may be because at high concentrations, the flexible blades experience resistance close to their maximum capacity, resulting in poorer deformation performance.

### 3.2. Suitable Velocity Ratio

For stirred-tank bioreactors, increasing the stirring speed can directly enhance the degree of TKE and shorten the mixing time, thereby increasing the rate at which microorganisms take up nutrients and reducing the time required for fermentation. However, the strong shear stress caused by excessively high stirring speed has a significant impact on microbial activity, reduce cell viability, and decrease reaction efficiency [40]. Therefore, maintaining the velocity within the optimal range in the bioreactor is of great significance for improving the reaction efficiency.

With reference to the definition of the low-velocity zone, the regions where the velocity is relatively high, creating a velocity difference with the surrounding areas are defined as high-velocity zones. In this work, the high-velocity zone is the region where the velocity is greater than 0.75 m/s. At a velocity below 0.125 m/s, the efficiency of microbial cells in absorbing nutrients is limited. When the velocity exceeds 0.75 m/s, the strong shear stress will affect the survival of sensitive cells.

Within this velocity range, the hydrodynamic shear stress exhibits a mean value of 3.113 Pa and a maximum of 10.187 Pa. Specifically, the regions where shear stress remains below 5 Pa, 6 Pa, and 7 Pa account for 83.2%, 91.1%, and 95.5%, respectively. Previous studies have demonstrated that the shear stress induced by the selected velocity range falls within the tolerance limits of various microbial cells and fungi, ensuring a high degree of biocompatibility [41]. Therefore, in this section, the velocity range of 0.125~0.75 m/s is defined as the suitable velocity range (*v*_s_) to comprehensively measure mixing efficiency and the impact of shear stress. The percentage of the area occupied by suitable velocity on the PIV-captured section is defined as the suitable velocity ratio (*φ*). A suitable velocity ratio difference (Δ*φ*) between rigid and flexible impellers is employed to comprehensively evaluate the performance of the two impeller types, while the larger the value, the more pronounced the balancing mixing efficiency and shear force advantage of the flexible impellers. Δ*φ* is calculated according to Equation (11) as follows:

(11) $$\Delta \varphi =\varphi_{F}-\varphi_{R}$$

**Figure 7:**
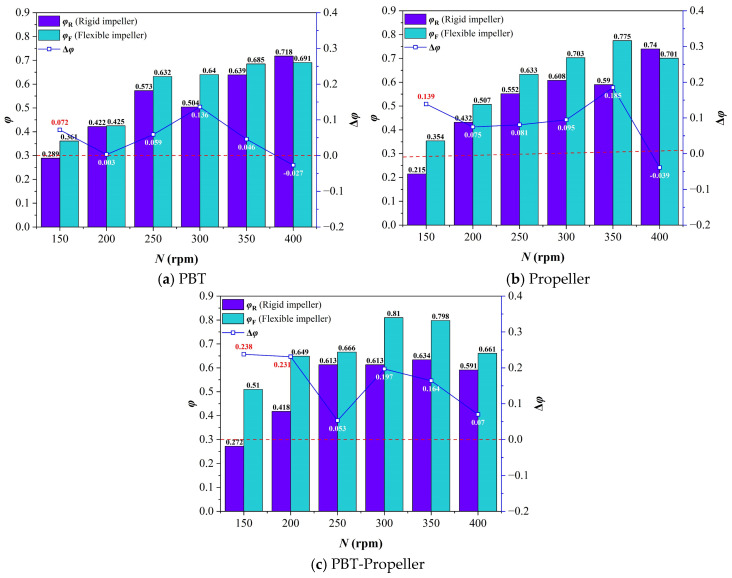
Suitable velocity ratio in the bioreactor (*ω*_1_ = 0 wt%).

As shown in Figure 7, when *ω*_1_ is 0 wt%, with the increase in stirring speed, the suitable velocity ratio in the bioreactor under the three impeller types shows a trend of increasing rapidly at low stirring speeds and increasing slightly or decreasing slightly at high stirring speeds. The average of the suitable velocity ratio is 57.3%. This is because the increase in stirring speed leads to a rise in the kinetic energy input into the bioreactor, resulting in an overall improvement in the velocity distribution. However, excessively high stirring speed will lead to an excessively high ratio of peak velocity, which in turn causes the growth of the suitable velocity ratio to slow down or even decrease slightly. In addition, the suitable velocity ratio under the double-layer impeller is generally higher than that of the single-layer. This further proves the advantages of multi-layer impellers in bioreactors with a relatively high aspect ratio.

By comparison, it is found that the overall suitable velocity ratio under the flexible impellers is better than that under rigid impellers, with an average increase in 9.9%. As *N* ≤ 350 rpm, the performance of flexible impellers is better than that of rigid impellers. However, when *N* is 400 rpm, the effect of flexible impellers weakens and can even be worse than that of rigid impellers.

**Figure 8:**
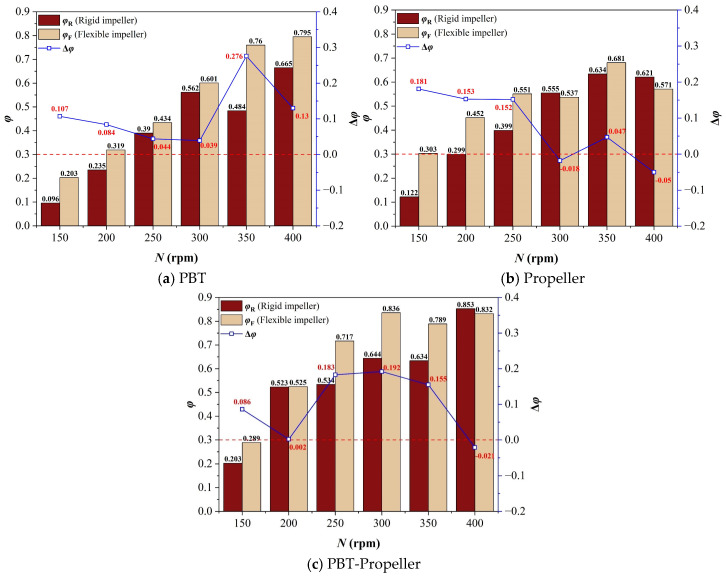
Suitable velocity ratio in the bioreactor (*ω*_1_ = 0.5 wt%).

As shown in Figure 8, when *ω*_1_ increases to 0.5 wt%, the suitable velocity ratio in the bioreactor maintains a trend of increasing rapidly at low stirring speeds and increasing slightly or decreasing at high stirring speeds. The average of the suitable velocity ratio is 51.8%. Compared with when *ω*_1_ is 0 wt%, the suitable velocity ratio at low stirring speeds decreases, and the downward trend is more significant for a single-layer impeller. This is because when *ω*_1_ increases, a single-layer impeller cannot form effective axial circulation at low stirring speeds, and the low-velocity zones inside the bioreactor occupy most of the area. Due to the arrangement of two impellers, double-layer impellers can agitate more areas inside the bioreactor.

Through comparison, it is found that when *ω*_1_ increases to 0.5 wt%, the overall suitable velocity ratio in the bioreactor decreases 5.5% compared with that when *ω*_1_ is 0 wt%. However, flexible impellers still maintain a certain advantage over rigid impellers in terms of kinetic energy dispersion, with the average suitable velocity ratio being 9.7% higher. Moreover, this advantage is more obvious at medium and low stirring speeds (when *N* ≤ 300 rpm).

**Figure 9:**
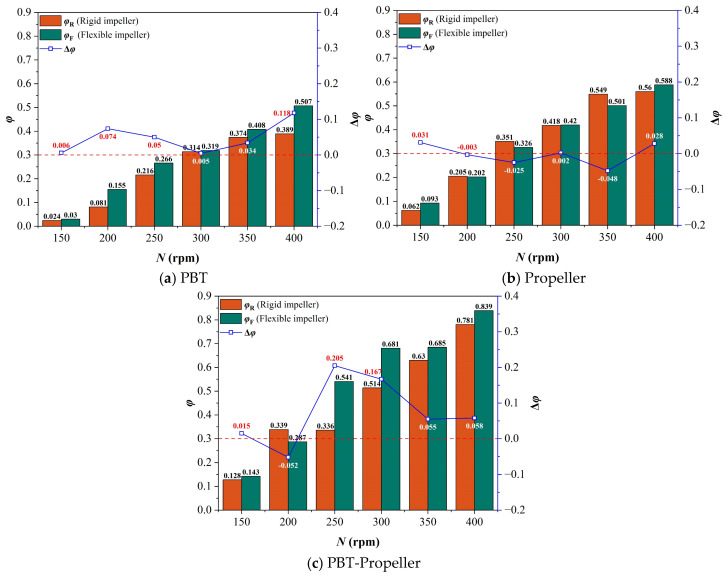
Suitable velocity ratio in the bioreactor (*ω*_1_ = 1 wt%).

As shown in Figure 9, when *ω*_1_ continues increasing to 1 wt%, the suitable velocity ratio in the bioreactor shows an obvious downward trend, with the average suitable velocity ratio being only 36.9%. The average suitable velocity ratios under PBT, propeller, and PBT-propeller (rigid and flexible impellers) are 0.445, 0.491, 0.684, 0.580, 0.869, and 0.776 times the value when *ω*_1_ is 0 wt%.

By comparison, it is found that when *ω*_1_ is 1 wt%, for a single-layer impeller, the suitable velocity ratios under the two sets of impellers (rigid and flexible) are basically the same. Compared with rigid impellers, the average ratio of suitable velocity under flexible impellers is just 2.3% higher. For double-layer impellers, flexible impellers still have a relatively obvious advantage over rigid impellers, with the average ratio of suitable velocity under flexible impellers being 7.5% higher. This may be because when the *ω*_1_ increases sufficiently, the liquid-phase resistance becomes excessively high. A single-layer impeller can no longer provide sufficient mixing power, and the effect of simply relying on increasing stirring speed to eliminate low-velocity zones is limited.

### 3.3. Mixing Time

Mixing time refers to the time consumed from the moment when materials are added to the reactor until the materials are uniformly dispersed in the reactor. As a complex fluid mechanics parameter, it is affected by multiple factors such as the structure of the stirring impellers, the shape of the reaction vessel, the physical properties of the liquid phase, and the ambient temperature. For bioreactors, the mixing of fluids is of great importance. It measures the efficiency of the reactor in supplying nutrients to microbial cells [42]. In this section, the color change method is used to conduct visualization experiments on the structural evolution of the flow field during the mixing process, and the mixing time under different working conditions is measured. The mixing time difference (∆*t*) between rigid and flexible impellers is used to compare the mixing performance of the two impeller types, while the larger the value, the more pronounced the mixing advantage of the flexible impellers.

**Figure 10:**
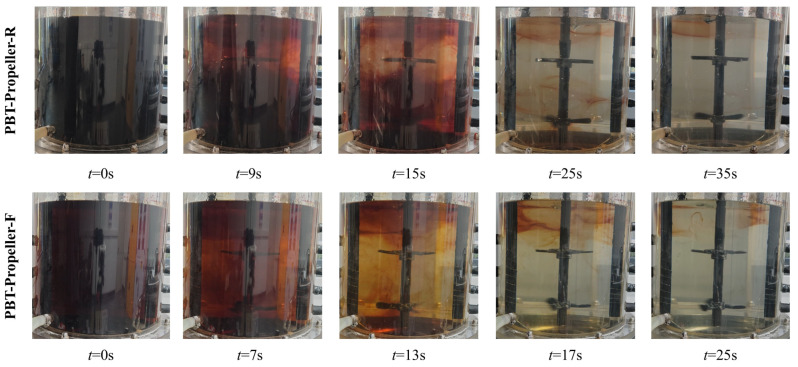
Visual images of different mixing stages (*ω*_1_ = 1 wt%, *N* = 250 rpm).

As shown in Figure 10, iodine solution with *ω*_2_ of 30 wt% was added to the transparent bioreactor, resulting in a uniformly stable reddish-brown color. A slight excess of sodium thiosulfate solution with *ω*_3_ of 27 wt% was continuously added, and the reddish-brown color gradually faded to colorless.

In the bioreactor with rigid impellers, discoloration first occurs near the installation position of the upper impeller, followed by discoloration near the lower stirring impeller. An obvious stratification phenomenon occurs. There are mixing dead zones at the middle position of the two-level impeller installation, at the bottom of the vessel, and at the liquid surface. As the stirring speed further increases, the mixing dead zones at the middle position of the two-level impeller installation and at the bottom of the vessel are eliminated. However, the mixing dead zone at the liquid surface persists.

In the bioreactor with flexible impellers, discoloration starts almost simultaneously from the position of the upper impeller to that of the lower impeller. The stratification phenomenon is not obvious. The time consumed to eliminate the mixing dead zones is shorter. This may be because at low stirring speeds, the rigid blades can only disturb a small area when stirring. Thus, there is an obvious mixing dead zone between the two layers of impellers. With the increase in stirring speed, most of the mixing dead zones are eliminated. However, the area near the liquid surface is difficult to fade completely due to the excessively high liquid concentration and the weak agitation effect of the stirring impellers. When flexible impellers are in operation, the flexible blades can affect a larger area due to their ability to undergo adaptive deformation, resulting in a more obvious fading effect.

**Figure 11:**
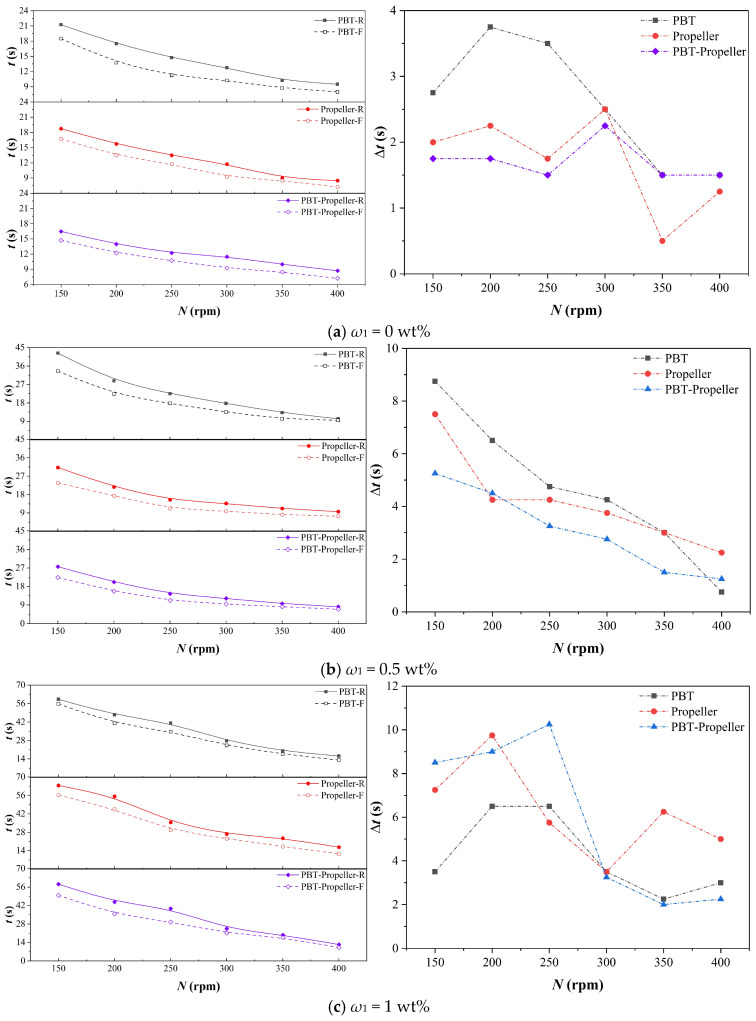
Mixing time in the bioreactor.

As shown in Figure 11, the mixing times under three different concentrations all show a linear decreasing trend with the increase in stirring speed. The mixing time under flexible impellers is generally shorter than that under rigid impellers. At the same concentration, the optimization effect of flexible blades is more significant at low speeds and they can reduce more mixing time. With the stirring speed increases, this optimization effect gradually diminishes, until the mixing time under rigid and flexible impellers is basically the same.

When *ω*_1_ increases from 0 wt% to 0.5 wt% and 1.0 wt%, the average values of mixing time saved by flexible impellers are 2.1 s, 3.3 s, and 5.4 s. The time saved increases in sequence, but the saving ratios are all around 15%. This is because as the concentration increases, the base value of the required mixing time becomes larger. Although the time saved by the flexible impellers increases, there is no significant change in the saving ratio.

### 3.4. Power Consumption

Stirred equipment has the advantages of high mixing efficiency, strong adaptability and simple structure. It occupies a large market share in the production of products such as pharmaceuticals, beverages and food. Currently, optimizing stirred equipment to reduce power consumption has become a research focus. By comparing the power consumption of rigid and flexible impellers, the potential of flexible impellers in consumption reduction is explored. The larger the power consumption per unit volume difference (Δ*P*_V_) between rigid and flexible impellers, the more pronounced the energy-saving advantage of the flexible impellers.

**Figure 12:**
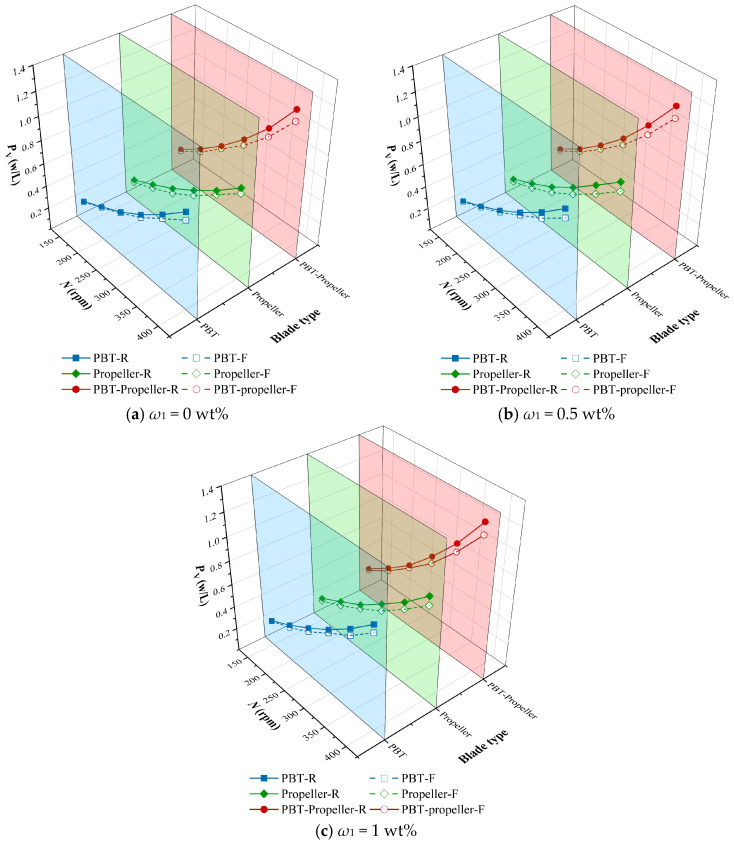
Power consumption in the bioreactor.

As shown in Figure 12, *P*_V_ increases exponentially with the increase in stirring speed. At the same working conditions, *P*_V_ is the highest under the PBT-propeller (with an average of 0.674 W/L), and the lowest under the propeller (with an average of 0.471 W/L). The *P*_V_ of flexible impellers is generally lower than that of rigid impellers, with an average *P*_V_ saving of 7.49%. Moreover, this reduction effect becomes more obvious when the concentration and stirring speed are higher. This may be because flexible impellers can undergo adaptive deformation based on the liquid-phase resistance they experience, thereby reducing the resistance that acts directly on the impeller surface.

Table 3 shows the power consumption per unit volume difference (Δ*P*_V_, W/L) between rigid impellers and flexible impellers under different working conditions. When at the same *ω*_1_, as the stirring speed increases, the Δ*P*_V_ gradually increases. When the stirring speed is the same, as *ω*_1_ increases from 0 wt% to 0.5 wt%, the average value of Δ*P*_V_ increases from 0.0371 W/L to 0.0458 W/L, and the average reduction ratio increases from 6.9% to 8.1%. When *ω*_1_ continues to increase to 1 wt%, the average value of Δ*P*_V_ decreases to 0.0444 W/L, and the average reduction ratio also drops to 7.49%. As *ω*_1_ continues to increase, the liquid-phase resistance borne by the flexible blades gradually approaches the maximum limit they can withstand, and the elastic material can no longer produce appropriate deformation. Thus, the effect of the flexible impellers weakens.

## 4. Conclusions

In stirred-tank bioreactors, the motion of the impellers exerts a significant influence on both mixing efficiency and microbial activity. This work proposes a performance detection method coupling PIV and image recognition, aiming to experimentally verify the influence of flexible blades on the flow characteristics of the system under different liquid concentrations, stirring speeds and impeller types. The main conclusions are as follows:

Velocity distribution shows an upward trend as *N* increases. At low *ω*_1_, increasing *N* can eliminate 50% low-velocity zones. Double-layer impellers can solve low-velocity zones at the liquid surface and the bottom of the tank under high *ω*_1_. Compared with rigid impellers, flexible impellers can reduce the peak velocity proportion and decrease the low-velocity zones by an average of 7.7%, which leads to a more uniform velocity distribution.

(1) An increase in *N* within the appropriate range will raise the suitable velocity ratio. However, excessively high *N* will lead to an overly high proportion of peak velocity, resulting in a subsequent decrease in the suitable velocity ratio instead. The increase in *ω*_1_ intensifies the difficulty of impeller agitation, resulting in a decrease in the overall velocity distribution. Compared with rigid impellers, the suitable velocity ratio under flexible impellers is 7.88% higher.
(2) A distinct stratification phenomenon appears in the flow field structure under rigid impellers. Under the same conditions, flexible impellers can agitate a wider area and the stratification phenomenon is effectively weakened. The agitation near the liquid surface is relatively weak, making it difficult to eliminate the mixing dead zones. Mixing time shows a downward trend with *N* increases. The average mixing time under flexible impellers is 15% less than that under rigid impellers.
(3) *P*_V_ shows an exponential growth trend with the increase in *N*. Flexible impellers have 7.49% lower *P*_V_ than rigid impellers. Under the same operating conditions, *P*_V_ is the lowest under the propeller and highest under the PBT-propeller. As *N* increases, Δ*P*_V_ shows an upward trend. When *ω*_1_ increases from 0 wt% to 0.5 wt% and 1 wt%, the average values of Δ*P*_V_ are 0.0371 W/L, 0.0458 W/L, and 0.0444 W/L, and the average reduction ratios are 6.9%, 8.1%, and 7.49%.

This study verifies the application potential of flexible impellers in fermentation. The fluid–structure interaction effectively reduces power consumption, shortens mixing time, and increases the suitable velocity ratio. For *ω*_1_ ≤ 1 wt%, favorable mixing can be achieved by adopting the PBT-propeller with a rotating speed of 300 rpm. For *ω*_1_ 1 wt%, the mixing mechanism is equally applicable, while flexible impellers can also be adapted to various conditions by adjusting different blades.

## Figures and Tables

**Table 1:** Parameters of experimental instruments.

Name	Type	Manufacturer	Accuracy
Electronic balance	JM	Kyoto Electronics Manufacturing Co., Ltd., Kyoto, Japan	0.01 g
Torque sensor	ZHO7-G	Testing & Control Technology Co., Ltd., Beijing, China	0.3%
High-speed camera	X150-256G (Revealer)	HF Agile Device Co., Ltd., Hefei, China	/
Laser	MGL-W-532-20W	Changchun New Industries Optoelectronics Technology Co., Ltd., Changchun, China	/
Rotational viscometer	MCR 102	Anton Paar (Shanghai) Trading Co., Ltd., Shanghai, China	/

**Table 2:** Experimental operating conditions.

Case	Installation Location	Blade Type	Liquid Environment *ω*_1_ (wt%)	Rotation Speed *N* (rpm)
A1	Middle (*H*_2_)	PBT-R	0 (Water)	150, 200, 250, 300, 350, 400.
A2		PBT-F		
A3		Propeller-R		
A4		Propeller-F		
A5	Top and Bottom (*H*_1_ and *H*_3_)	PBT-Propeller-R		
A6		PBT-Propeller-F		
B1	Middle (*H*_2_)	PBT-R	0.5 (CMC solution)	150, 200, 250, 300, 350, 400.
B2		PBT-F		
B3		Propeller-R		
B4		Propeller-F		
B5	Top and Bottom (*H*_1_ and *H*_3_)	PBT-Propeller-R		
B6		PBT-Propeller-F		
C1	Middle (*H*_2_)	PBT-R	1 (CMC solution)	150, 200, 250, 300, 350, 400.
C2		PBT-F		
C3		Propeller-R		
C4		Propeller-F		
C5	Top and Bottom (*H*_1_ and *H*_3_)	PBT-Propeller-R		
C6		PBT-Propeller-F		

**Table 3:** Power consumption per unit volume difference in different operating conditions.

*N* (rpm)	*ω*_1_ (wt%)	Δ*P*_V_ (W/L)		
		PBT	Propeller	PBT-Propeller
150	0	0.0052	0.0251	0.0162
	0.5	0.0079	0.0283	0.0162
	1	0.0052	0.0251	0.0157
200	0	0.0098	0.0356	0.0286
	0.5	0.0119	0.0426	0.0272
	1	0.0216	0.0377	0.0251
250	0	0.0105	0.0445	0.0270
	0.5	0.0166	0.0515	0.0366
	1	0.0340	0.0358	0.0236
300	0	0.0251	0.0461	0.0513
	0.5	0.0230	0.0607	0.0555
	1	0.0304	0.0597	0.0576
350	0	0.0330	0.0354	0.0708
	0.5	0.0501	0.0745	0.0769
	1	0.0562	0.0598	0.0696
400	0	0.0670	0.0447	0.0921
	0.5	0.0740	0.0768	0.0949
	1	0.0670	0.0754	0.1005

## Data Availability

The data that support the findings of this study are available from the corresponding author upon reasonable request. The data are not publicly available due to confidentiality obligations from industrial cooperation projects.

## Nomenclature

*B*	Component of blue in RGB color space: dimensionless
*D*_1_	Rigid impeller diameter, mm
*D*_2_	Diameter of the rigid part of flexible impeller, mm
*d*	Thickness of flexible impeller, mm
*dx*(*t*)	Displacement difference of tracer particles in x-direction, m
*dy*(*t*)	Displacement difference of tracer particles in y-direction, m
*G*	Component of green in RGB color space, dimensionless
*H*	Component of hue in HSV color space, dimensionless
*H*_0_	Liquid vertical height, mm
*H*_1_	Impeller installation position 1, mm
*H*_2_	Impeller installation position 2, mm
*H*_3_	Impeller installation position 3, mm
*K*	Consistency coefficient, Pa·s^*n*^
*l*	Length of flexible impeller, mm
*M*_1_	Load torque, N·m
*M*_0_	No-load torque, N·m
*N*	Stirring speed, rpm
*n*	Flow behavior index, dimensionless
*P*	Input power consumption, W
*P*_V_	Power consumption per unit volume, W/L
Δ*P*_V_	Power consumption per unit volume difference, W/L
*R*	Component of red in RGB color space, dimensionless
*r*	Radial displacement coordinate, m
*S*	Component of saturation in HSV color space, dimensionless
*T*	Tank diameter, mm
*t*	Mixing time, s
∆*t*	Mixing time difference between rigid and flexible impellers, s
*u_r_*	Radial instantaneous velocity, m/s
*u_z_*	Axial instantaneous velocity, m/s
*V*	Component of value in HSV color space, dimensionless
*V*_0_	Liquid volume, L
*v*_x_	Component velocity of tracer particles in x-direction, m/s
*v*_y_	Component velocity of tracer particles in y-direction, m/s
*v*_0_	Resultant velocity of tracer particles, m/s
*v*_s_	Suitable velocity, m/s
*z*	Axial displacement coordinate, m
*Greek Letters*	
$\dot{\gamma }$	Shear rate, 1/s
${\dot{\gamma }}_{rz}$	Meridional shear rate, 1/s
${\dot{\varepsilon }}_{rr}$	Radial normal strain rate, 1/s
${\dot{\varepsilon }}_{zz}$	Axial normal strain rate, 1/s
${\dot{\varepsilon }}_{\theta \theta }$	Tangential normal strain rate, 1/s
*θ*	Impeller tilt angle, °
*μ*_a_	Apparent viscosity, Pa·s
*τ*	Shear stress, Pa
*φ*	Suitable velocity ratio, dimensionless
*φ*_R_	Suitable velocity ratio of rigid impeller, dimensionless
*φ*_F_	Suitable velocity ratio of flexible impeller, dimensionless
Δ*φ*	Suitable velocity ratio difference, dimensionless
*ω*_1_	Concentration of CMC solution, wt%
*ω*_2_	Concentration of iodine solution, wt%
*ω*_3_	Concentration of sodium thiosulfate solution, wt%
